# DCFE-YOLO: A novel fabric defect detection method

**DOI:** 10.1371/journal.pone.0314525

**Published:** 2025-01-14

**Authors:** Lei Zhou, Bingya Ma, Yanyan Dong, Zhewen Yin, Fan Lu

**Affiliations:** Faculty of Computer and Software Engineering, Huaiyin Institute of Technology, Huai’an, Jiangsu, China; Government College University Faisalabad, PAKISTAN

## Abstract

Accurate detection of fabric defects is crucial for quality control in the textile industry. However, the task of fabric defect detection remains highly challenging due to the complex textures and diverse defect patterns. To address the issues of inaccurate localization and false positives caused by complex textures and varying defect sizes, this paper proposes an improved YOLOv8-based fabric defect detection method. First, Dynamic Snake Convolution is introduced into the backbone network to enhance sensitivity to elongated and subtle defects, improving the extraction of edge and texture details. Second, a Channel Priority Convolutional Attention mechanism is incorporated after the Spatial Pyramid Pooling layer to enable more precise defect localization by leveraging multi-scale structures and channel priors. Finally, the feature fusion network integrates Partial Convolution and Efficient Multi-scale Attention, optimizing the fusion of information across different feature levels and spatial scales, which enhances the richness and accuracy of feature representations while reducing computational complexity. Experimental results demonstrate a significant improvement in detection performance. Specifically, mAP@0.5 increased by 2.9%, precision improved by 3.5%, and mAP@0.5:0.95 rose by 2.3%, highlighting the model’s superior capability in detecting complex defects. The project is available at https://github.com/lilian998/fabric.

## Introduction

Fabric defect detection in the textile industry is a highly challenging task. Quality inspection of textiles is a critical stage in the production chain, significantly impacting product quality, pricing, economic efficiency, and market competitiveness of enterprises [1, 2]. Defects in fabrics not only affect the appearance, durability, and lifespan of products but can also reduce product prices by 45% to 65%, highlighting the critical importance of effective defect detection [3]. Therefore, timely and accurate detection of fabric defects is essential for ensuring product quality. Effective detection can prevent defective products from reaching the market, while also identifying potential issues early, enabling swift corrective action to minimize negative impacts on brand image and customer satisfaction.

Fabric defect detection involves identifying the location, type, and size of surface defects [4]. This process often takes place in complex production environments, where factors such as lighting variations, fabric textures, and fluctuating defect sizes create additional challenges. These factors not only complicate the detection process but also place high demands on the real-time performance of detection algorithms. In fast-paced, dynamic production environments, it is imperative that detection algorithms accurately and promptly identify fabric defects to maintain product quality.

Traditional methods of fabric defect detection mainly rely on manual visual inspection, which is inefficient, with a maximum inspection speed of only 12 meters per minute [5]. Studies indicate that the accuracy of manual defect detection ranges from 60% to 75% [6]. Furthermore, manual visual inspection is susceptible to inspector fatigue, experience, and subjective judgment, affecting the accuracy of detection results [7, 8].

The advancement of computer technology, particularly in artificial intelligence, has brought new opportunities for fabric defect detection. Deep learning-based object detection algorithms have made notable progress in this field. Lu and Huang [9] introduced the TADet detection model, which enhances performance by distinguishing defects from normal fabric textures and demonstrates strong robustness against noise. Cheng et al. [10] developed the SCUnet model, which integrates convolutional downsampling, depthwise separable convolutions, and a cross-parallel loss function to extract surface features from fabric images for defect localization, addressing the issue of low recall rates. While these methods have improved fabric defect detection from various angles, challenges remain, particularly with high false positive and missed detection rates when dealing with defects that blend with the background. Additionally, extracting sufficient defect features remains difficult, and further optimization of algorithm accuracy is needed. To meet the growing demand for real-time defect detection in fabric inspection, algorithm efficiency is also a key priority. As a result, there has been increasing interest in optimizing single-stage object detection algorithms for faster, more precise defect detection.

Based on the advantages of YOLOv8n in detection speed and efficiency, this paper proposes an improved algorithm to address challenges such as inaccurate localization and false positives caused by complex fabric textures and variable defect sizes. The main contributions of this paper are as follows:

1. Firstly, the backbone network of this model incorporates Dynamic Snake Convolution (DSConv) [11], which dynamically adjusts the convolutional kernel shape to flexibly capture elongated and subtle defect regions. By adaptively reshaping the kernel, DSConv enhances the model’s sensitivity to irregularly shaped defects, particularly fine linear ones, thus improving its precision in detecting complex defects.
2. Secondly, after the Spatial Pyramid Pooling Fast (SPPF) layer in the backbone network, a Channel Priority Convolutional Attention mechanism [12] is introduced. This mechanism prioritizes critical channel information by focusing on the most significant features within the channel dimension, leading to more effective feature extraction. Additionally, the integration of a multi-scale structure strengthens the model’s ability to capture spatial relationships, enabling it to detect defect regions of varying sizes simultaneously. This strategy effectively highlights important feature regions, enhancing both detection accuracy and robustness.
3. Finally, in the neck network, Partial Convolution (PConv) [13] and Efficient Multi-scale Attention (EMA) [14], based on cross-dimensional learning, are employed. PConv helps avoid processing irrelevant information, while cross-dimensional interactions improve the model’s ability to fuse information across different feature levels and spatial scales. This results in richer and more accurate feature representations while reducing both the number of parameters and computational complexity.

## Related work

Fabric defect detection algorithms can be broadly classified into two categories: traditional algorithms and deep learning-based algorithms [15].

### Traditional methods

Traditional image processing methods for fabric defect detection include structural methods, statistical methods, spectral analysis methods, and model analysis methods [16]. Among them, Shi et al. [17] proposed a fabric defect detection method based on low-rank decomposition of gradient information and structured graph algorithms. Yapi et al. [18] introduced a novel redundant contourlet transform to partition images into defective and defect-free regions. Anandan et al. [19] combined GLCM and curvelet transform (CT) to extract feature vectors of fabric defects, enhancing the saliency of defect features. Experimental results demonstrated the effectiveness of this method compared to approaches based on GLCM and wavelets. C.L. Li et al. [20] proposed a fabric defect detection method based on texture descriptors and low-rank decomposition models, which exhibited high detection accuracy and adaptive capability.

Traditional image processing methods for fabric defect detection rely on handcrafted features, which struggle to capture complex patterns and subtle defects. Additionally, these methods are highly susceptible to variations in lighting, texture interference, and background noise, leading to a decline in detection accuracy. With the emergence of convolutional neural networks, deep learning-based detection methods have addressed the shortcomings of traditional approaches and demonstrated significant advantages in practical industrial applications, thus being widely adopted in the field of fabric defect detection.

### Deep learning-based methods

Deep learning-based object detection can be categorized into two main types based on the detection approach, one is the two-stage object detection method based on region proposals, with typical models including R-CNN [21], Fast R-CNN [22], Faster R-CNN [23], and Mask R-CNN [24]. One approach involves generating candidate regions using a region proposal network, followed by classification and regression. While this method offers high accuracy, it is relatively slow. An alternative is single-object detection based on regression algorithms, with prominent models including the SSD [25] and YOLO [26–31]series. These methods treat object detection as a regression problem and perform both object classification and bounding box regression in a single network. The output vector contains both the class labels and the position information of the objects. This approach is characterized by lower computational complexity, faster detection speeds, and suitability for real-time applications. Ouyang et al. [32] proposed a fabric defect detection method based on activation layer embedded CNN, achieving a 92% accuracy in detecting complex fabrics, but only an 80% accuracy for small defects. Chen et al. [33] combined Gabor filters with Faster R-CNN, selecting optimal Gabor parameters using genetic algorithms to effectively address texture interference and significantly improve the accuracy of fabric defect recognition, although detection speed decreased correspondingly. Zhang et al. [34] introduced a channel attention mechanism into MobileNetV2-SSDLite to highlight defect features and suppress background noise features. They also redefined the loss function using Focal Loss to overcome the imbalance between defect and background candidate boxes. Jing et al. [35] applied K-Means clustering to the morphology of fabric defects in YOLOv3, selecting 12 scale prediction boxes for accurate shape prediction. They optimized the feature extraction network using feature fusion techniques to reduce false positives. Although this method has a fast detection speed, its detection accuracy is relatively low. Liu et al. [36] enhanced the performance of the YOLOv4 model by redividing anchor points, combining CLAHE for contrast enhancement, and using soft pool to improve the SPP structure. Zhu et al. [37] proposed the E-YOLOv5 network, which strengthened the extraction of fabric defect features and accelerated model convergence by adding dual-level cascaded attention modules and ghost shuffle convolutions to the YOLOv5 architecture, thereby improving detection accuracy. However, it performs poorly in complex backgrounds. Liu et al. [38] proposed a Feature Purification Fusion (FPF) structure based on YOLOv5, which includes a Semantic Information Supplementation Strategy (SIS) and a Detail Information Supplementation Strategy (DIS). The FPF enhances the network’s ability to detect small defects and effectively alleviates the aliasing effect that arises during feature fusion. However, the computational and parameter overhead of the improved model has significantly increased. Li et al. [39] introduced the YOLO-CA network, which optimizes the model using dual cross attention modules and cross-stage soft pooling feature pyramid modules. This optimization enhances the model’s ability to capture contextual information between fabric defects and backgrounds, improving localization accuracy and reducing the miss detection rate. Nonetheless, there is still room for improvement in recognizing similar defect categories. Liu et al. [40] proposed the PRC-Light YOLO, which replaced part of Conv with PConv to optimize feature extraction, expanded the receptive field using RFB pyramids and CARAFE upsampling operators, and used HardSwish to minimize memory access on the YOLOv7 basis. However, there are limitations such as unified background, limited quantity, and insufficient diversity of defect types.

While deep learning-based fabric defect detection methods have shown promising results, certain challenges remain. For instance, existing algorithms often struggle to differentiate between normal textures and defects in complex backgrounds, resulting in a higher rate of false positives. Additionally, the model’s performance is less than ideal when addressing multi-scale defects and the imbalance in candidate box numbers. To tackle these issues, this paper proposes an improved algorithm designed to reduce false positives and false negatives in multi-scale fabric defect detection under complex backgrounds, thereby enhancing the model’s accuracy.

## Methods

In fabric defect detection, fabric images typically contain extensive textures and details, with defects varying in size, shape, and location. Given the high real-time requirements of fabric defect detection tasks, this paper proposes an improved model DCFE-YOLO, which has been improved based on YOLOv8n as follows.

First, DSConv is integrated into the backbone network, forming DSC_Bottleneck within the Bottleneck structure, which is then used to develop the DSC_C2f model. This enhances the capability to extract defect features, particularly focusing on elongated and weak defect regions, thereby improving detection accuracy.

Second, a CPCA attention mechanism layer is added after the SPPF layer. CPCA employs a Multi-Scale structure to boost the convolutional operations’ ability to capture spatial relationships. By utilizing Multi-Scale depth convolution modules, it effectively extracts spatial relationships while preserving channel priors, thus focusing on information channels and key areas.

**Fig 1:**
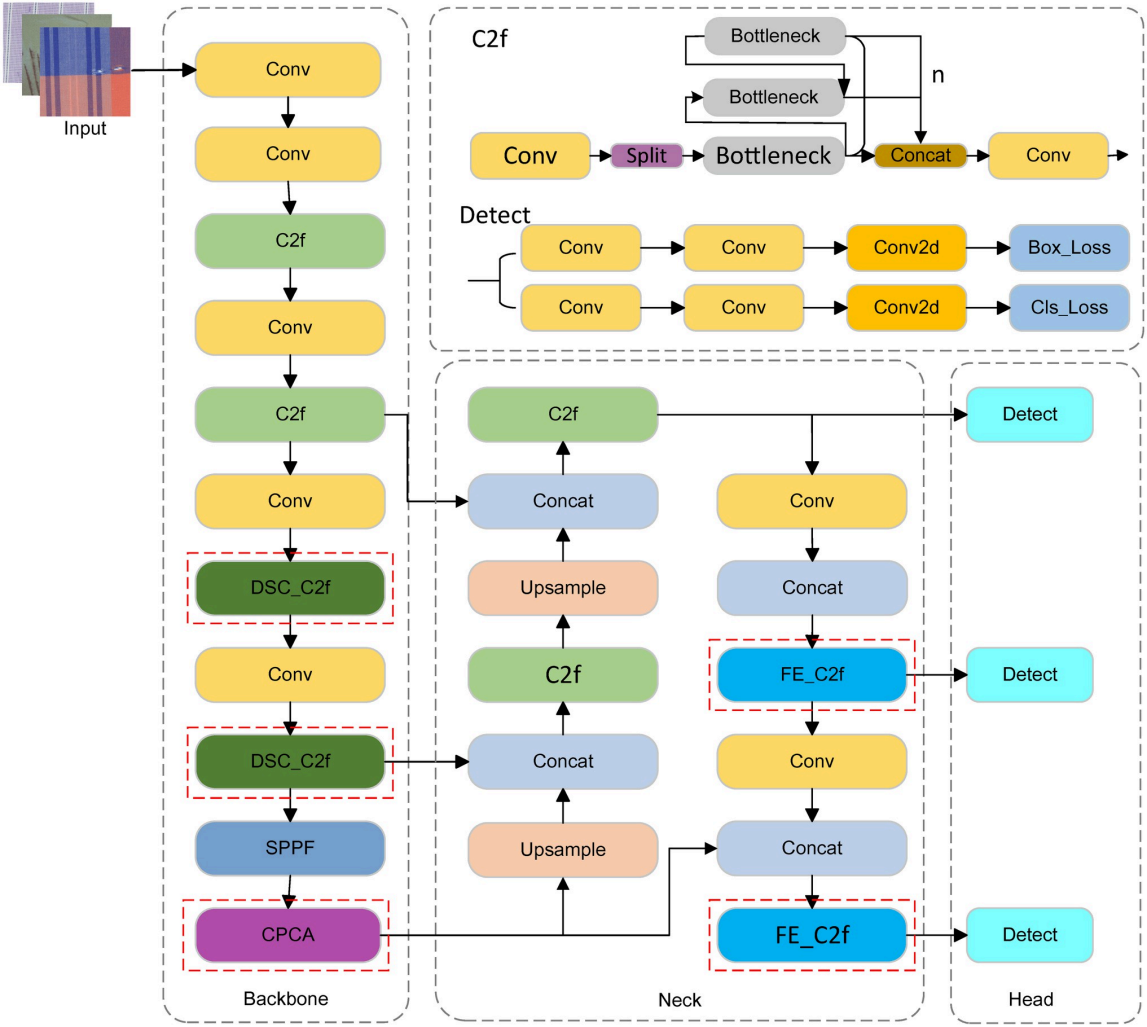
Improved DCFE-YOLOv8 algorithm.

Lastly, in the neck network, the FE_Block, constructed using Pconv and EMA, replaces the Bottleneck structure of C2f. The overall architecture of the improved model is illustrated in Fig 1.

### DSC_C2f module

**Fig 2:**
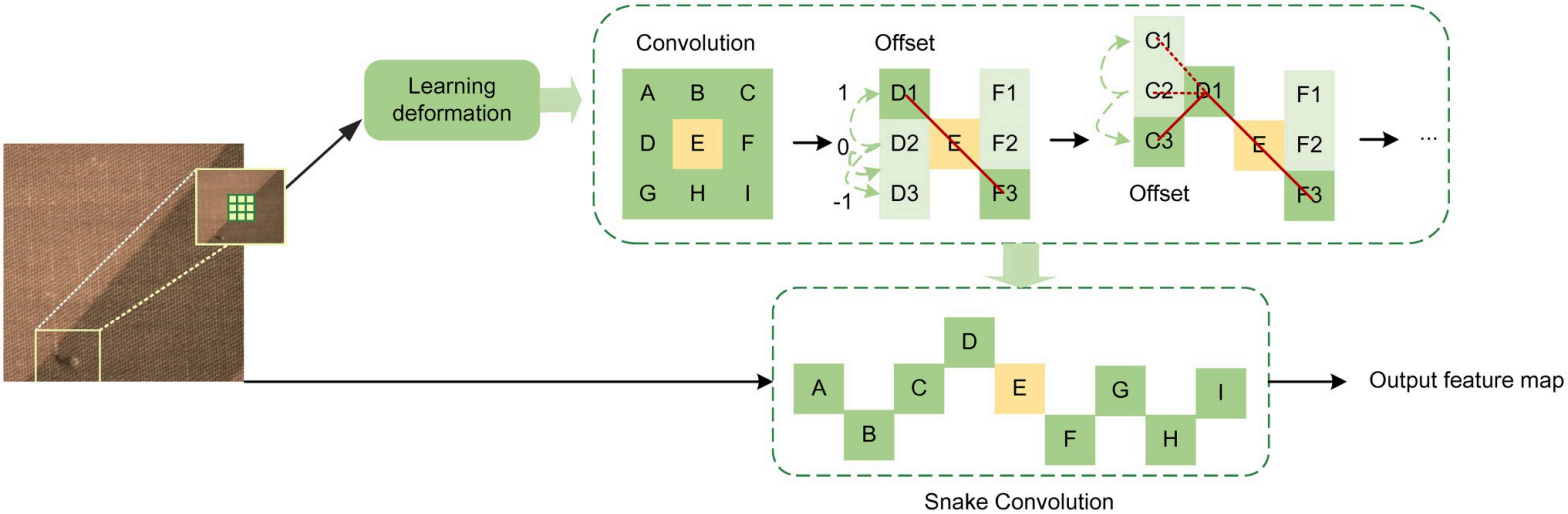
Overall structure of DSConv.

In fabric defect detection, the diversity of defects, including variations in size, shape, position, and orientation, often makes it challenging for standard convolution operations to accurately capture defect details or only capture partial information. To further enhance the network’s learning capability, this paper integrates DSConv into the Bottleneck to form the DSC_Bottleneck, which is then used as a C2f module to create the DSC_C2f module. Dynamic Snake Convolution adapts the shape of the convolution kernels dynamically to the variations in the input features, thereby improving the network’s ability to represent fine, weak local features and complex, variable global patterns. The flexibility of DSConv enables the network to detect minute defects in fabric across different scales and directions, significantly improving defect detection accuracy. The complete framework of DSConv is illustrated in Fig 2.

In Fig 2, the mechanism first receives an image or feature map, which undergoes initial processing through standard convolutional layers to extract basic features. Next, the feature information enters the core phase of Dynamic Snake Convolution (DSConv)—deformation learning. In this phase, the network learns to predict offsets through specialized layers, enabling the convolutional kernels to dynamically adjust their sampling positions to accommodate spatial deformations in the image. Each kernel element position (e.g., D1, D2) is associated with an offset, allowing the kernel to adaptively change shape to more accurately align with specific regions. These offsets are not assigned randomly but are learned, reflecting the deformation characteristics of local features. Leveraging these offsets, the convolutional points (e.g., C1, C2) flexibly slide across the feature map along a snake-like trajectory, adjusting adaptively to capture spatial features. This results in a rich feature map that integrates local deformation information.

**Fig 3:**
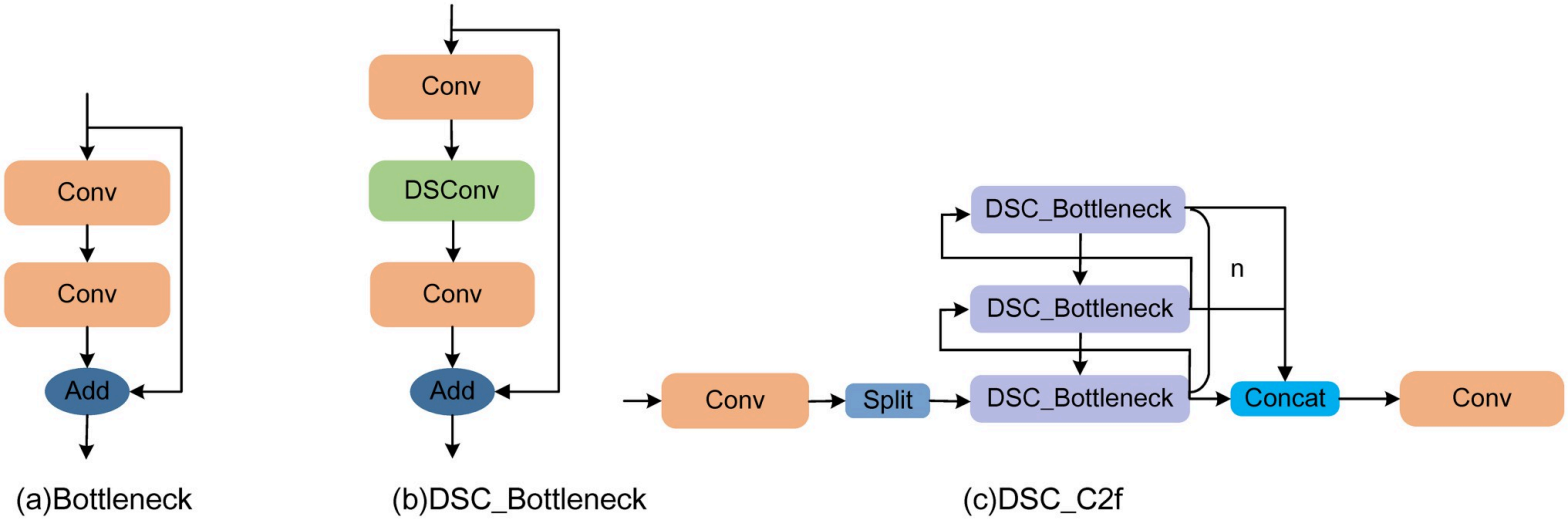
Improvements in the backbone network’s C2f module.

By applying DSConv to the C2f module in the backbone network, the network can capture minute defects in the fabric at different scales and directions, further extracting critical information within the network and consequently enhancing defect detection accuracy. The specific improvements are illustrated in Fig 3.

In Fig 3(a), the Bottleneck within C2f processes the input feature map through two convolution layers, and the features are fused through element-wise addition after these two layers. The skip connection helps gradients propagate more effectively.

In Fig 3(b), the improved Bottleneck structure, DSC-Bottleneck, adds a DSConv between the two standard convolution layers. Dynamically adjusts the positions of the convolution kernel by learning the deformation of the feature map. This kernel offset allows the network to adapt to local features in the image, enhancing the network’s ability to capture shape variations and irregular patterns.

In Fig 3(c), the improved C2f utilizes the improved DSC_Bottleneck to replace the original Bottleneck. The input feature map is first divided into two branches, with one part directly entering the series of DSC_Bottleneck modules while the other continues through the convolution layers. Finally, these two parts are merged through concatenation, followed by further processing through the final convolution layer, producing the final output feature map.

### CPCA module

**Fig 4:**
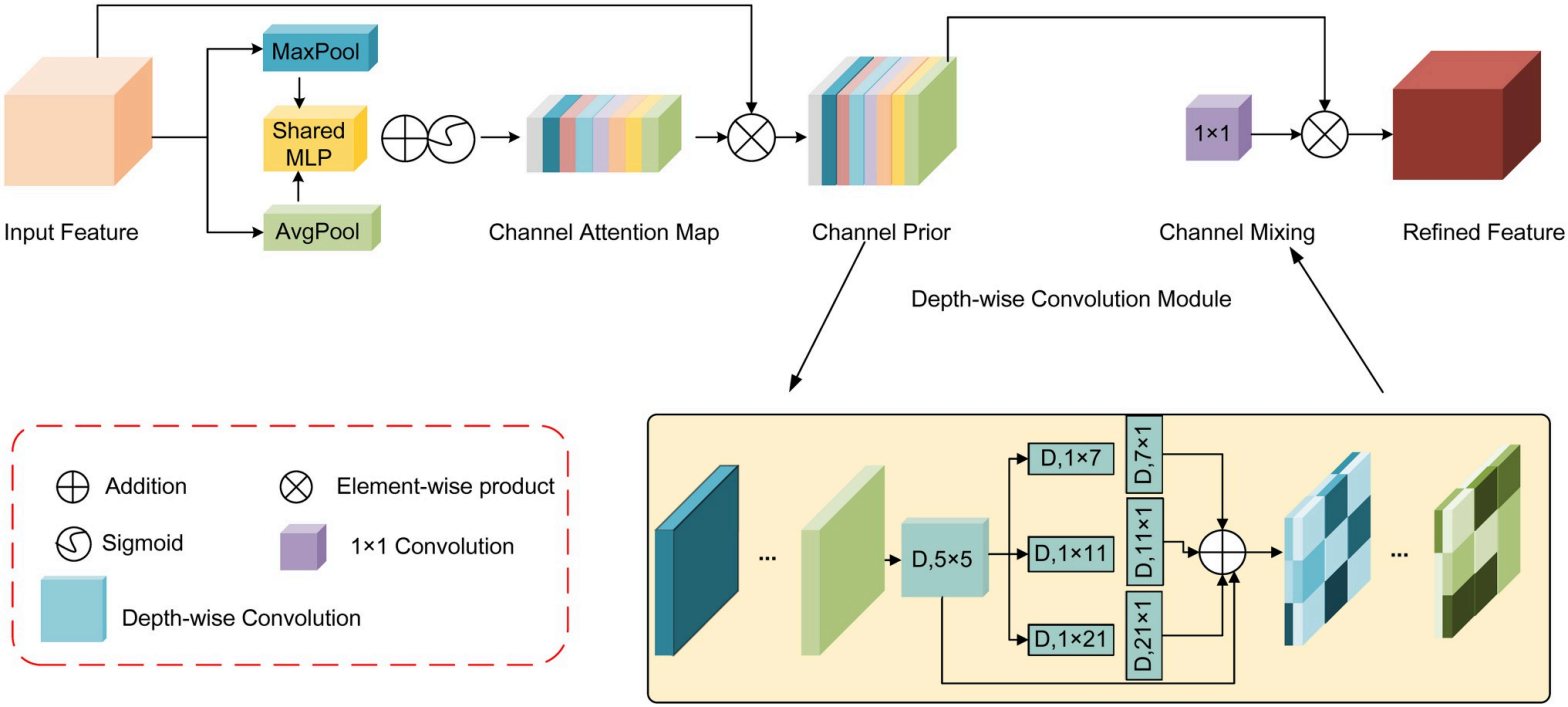
Structure of CPCA.

The CPCA utilizes a multi-scale depthwise separable convolution module to construct spatial attention, enabling dynamic distribution of attention weights across channel and spatial dimensions. This enhances the focus on information pathways and key areas. Fabric images typically contain rich textures and detailed features, making the application of spatial attention mechanisms crucial for accurately analyzing subtle texture variations within specific regions. Furthermore, fabric images exhibit a wide range of colors and brightness levels, necessitating the use of channel attention mechanisms to dynamically weight different channels. This helps in effectively identifying key features associated with various colors and lighting conditions. By incorporating the CPCA attention mechanism after the SPPF layer in the YOLOv8 backbone network, the feature map’s channel and spatial attention weights are adaptively adjusted, enhancing the feature representation capability. The structure of the CPCA is depicted in Fig 4.

As shown in the Fig 4, the CPCA structure integrates channel attention and spatial attention mechanisms to enhance feature extraction capabilities. Initially, the input feature map undergoes both average pooling and max pooling to aggregate spatial information from different perspectives, followed by the generation of a channel attention map C(F) using a shared Multi-Layer Perceptron (MLP). Subsequently, the input feature F is element-wise multiplied by the channel attention map C(F), creating a channel prior that effectively weights the important channels. Next, the channel prior is fed into a depthwise separable convolution module, which utilizes convolutional kernels of various sizes to extract multi-scale spatial information. The spatial feature maps from different scales are fused through summation, and a 1 × 1 convolution is applied for channel mixing, resulting in the generation of the spatial attention feature S(F). Finally, the spatial attention map is element-wise multiplied with the channel-attention-modulated features, producing the final feature map, thereby enhancing the network’s response to key spatial regions. The specific computational process of the CPCA mechanism is as follows:

$$\begin{array}{c} C(F)=\sigma (MLP\left(avgPool\left(F\right)\right)+MLP\left(MaxPool\left(F\right)\right) \end{array}$$ (1)

$$\begin{array}{c} F_{C}=C(F)⊗F \end{array}$$ (2)

$$\begin{array}{c} S(F)={Conv}_{1\times 1}(\sum_{i=0}3{\;Branch}_{i}(Dw(F))) \end{array}$$ (3)

$$\begin{array}{c} \hat{F}=S(F)⊗Fc \end{array}$$ (4)

Where *σ* is the Sigmoid activation operation, *C*(*F*)represents channel-wise attention features, and *S*(*F*) represents spatial attention features. Dw represents depth-wise convolution. Where *Branch*_*i*_, *i* ∈ {0, 1, 2, 3}, *Branch*_0_ represents identity connection.

### FE_C2f module

#### FE_Block

**Fig 5:**
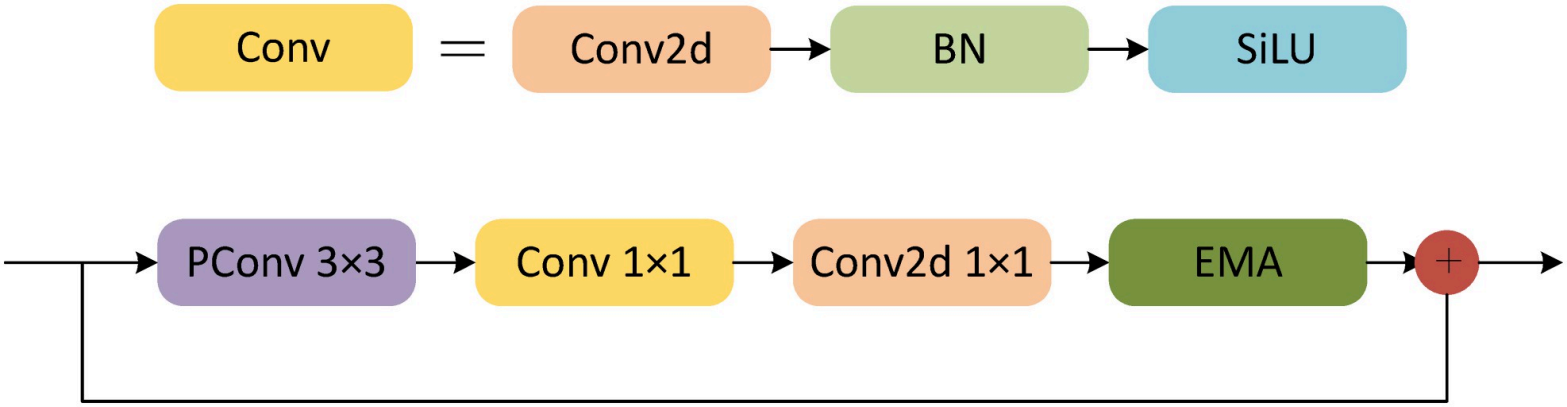
FE_Block.

The FE_Block, constructed using PConv and the EMA module from Fasternet, is depicted in Fig 5.

In the construction of the FE_Block, the input data is processed through PConv, where only 1/4 of the channels perform convolution operations to extract spatial features, while the remaining 3/4 of the channels remain unchanged to minimize unnecessary information redundancy. The output of the convolution operation is then concatenated with the unprocessed channels to further integrate the information. redundancy. The output of the convolution operation is then concatenated with the unprocessed channels to further integrate the information. Next, a Conv module is applied, which involves a 1 × 1 convolution, normalization, and activation functions to double the number of channels in the output feature map from the previous layer. This helps maintain feature diversity and reduce latency. Additionally, the 1 × 1 convolution is used for dimensional adjustment. Another 1 × 1 convolution is then applied for dimensionality reduction, ensuring the number of channels remains consistent with the input. This is followed by the addition of the EMA attention module to encode global information, achieve spatial information aggregation, and establish short-term and long-term dependencies. This results in multi-scale feature representation, enhancing the understanding of defect target context information.

**Fig 6:**
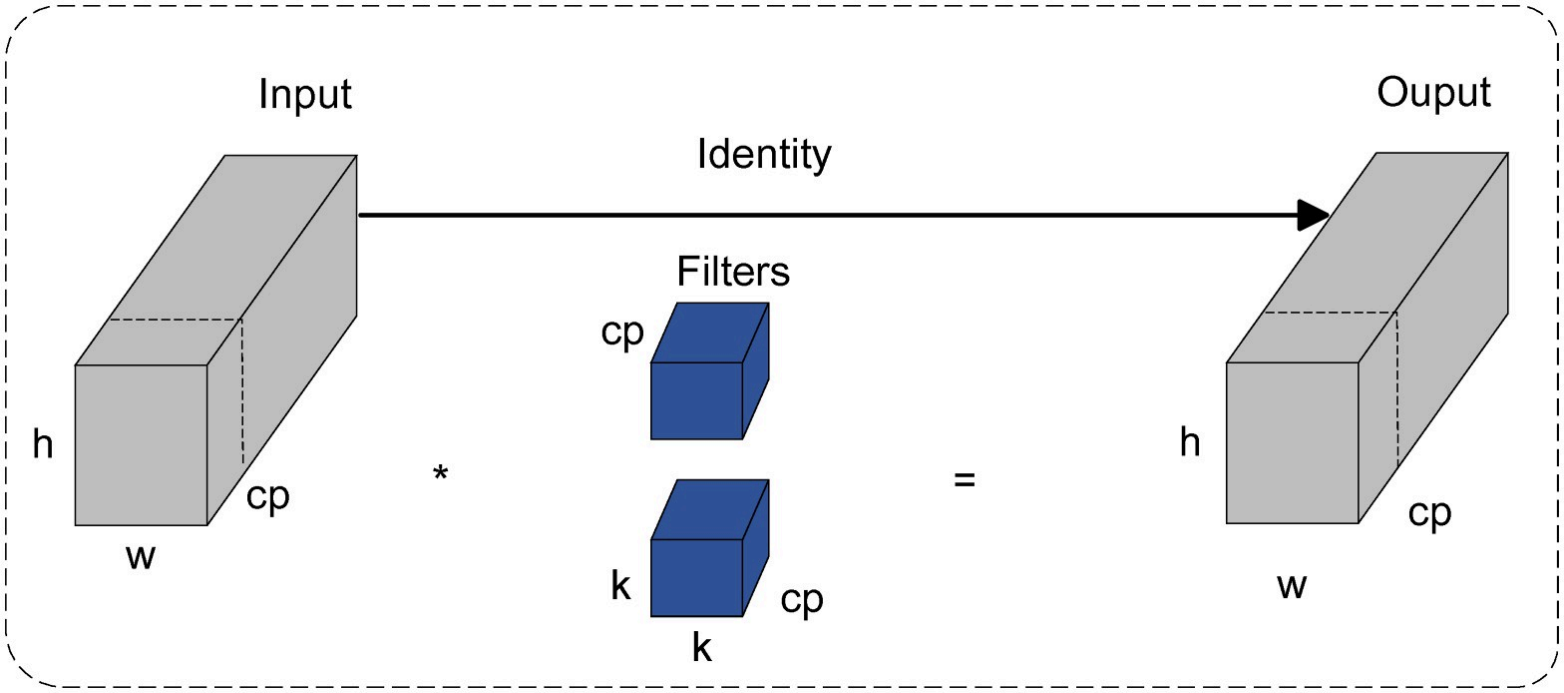
PConv structure.

The use of PConv makes the model more efficient at extracting spatial information while reducing computational complexity and memory usage. The PConv structure is illustrated in Fig 6.

The PConv method uses filters applied to the input image. For input feature maps with dimensions h × w × C the convolution operation only affects selected input channels, ignoring other channels to reduce computational demands. Here, h, w, and k represent the height, width, and kernel size of the feature map, respectively. Additionally, to minimize memory access times, especially when serialization or regular access patterns are needed, PConv can focus on computing the first or last continuous channels of the feature map. This helps reduce data loading and processing time, further lowering resource consumption.

**Fig 7:**
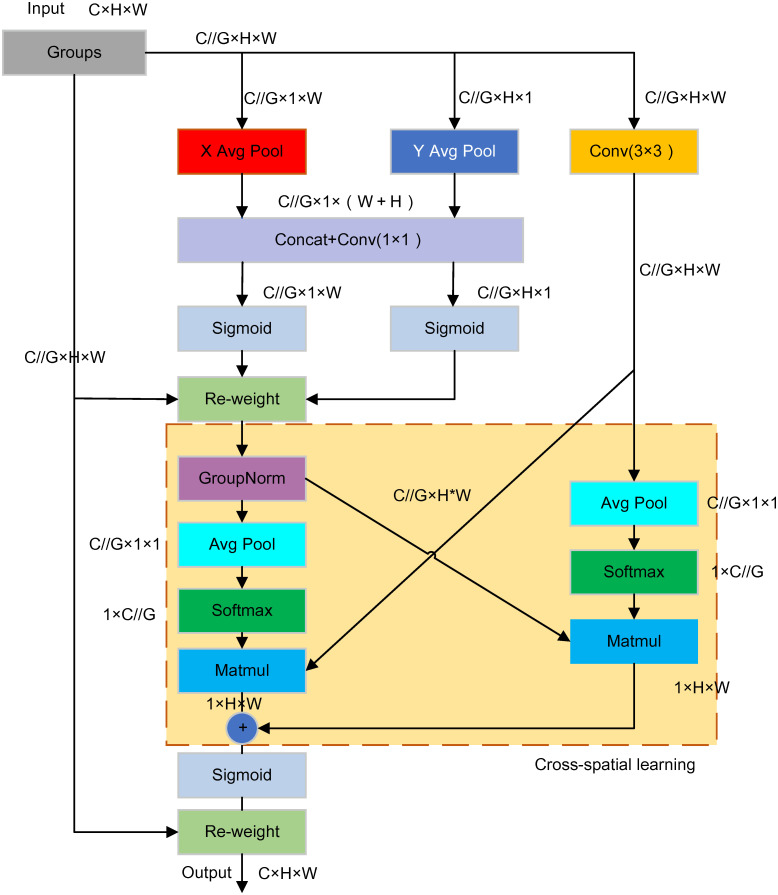
Overall structure of EMA attention mechanism.

The EMA attention mechanism used in the FE_Block is a new efficient multi-scale attention module. This module employs strategies such as multi-scale parallel sub-networks to model features of different channels while learning more effective channel descriptions while maintaining pixel-level attention. The overall structure of the EMA attention mechanism is shown in Fig 7.

The EMA attention mechanism refines feature processing by utilizing two parallel 1 × 1 convolution branches and a 3 × 3 convolution branch. The 1 × 1 branches perform global average pooling along the horizontal and vertical axes, capturing global information, which is then concatenated and further processed via a shared 1 × 1 convolution. In parallel, the 3 × 3 convolution branch focuses on capturing both local and contextual information, with its output also undergoing global average pooling to distill spatial information. The outputs from the 1 × 1 and 3 × 3 branches are subsequently fused to produce a hybrid feature map. This map is then passed through a 1 × 1 convolution and a Sigmoid function to generate the final attention map. The attention map is element-wise multiplied with the input feature map, enabling the network to apply weighted emphasis to key regions, thereby enhancing feature representation.

#### FE_Block

**Fig 8:**
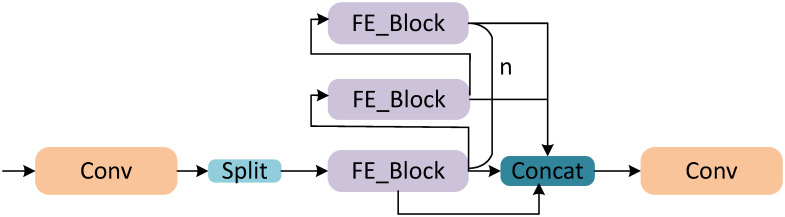
FE_C2f.

By replacing the Bottleneck in C2f with the FE_Block, the FE_C2f module is designed to enhance information fusion across different channels, retain more contextual information, and effectively reduce model parameters and floating-point computation. The structure of the FE_C2f module is shown in Fig 8.

In implementing the FE_C2f module, the input feature map is first processed by a convolutional layer, which expands the output channels to 2c. Next, the feature map is split into two parts. One part is fed into a series of n FE_Blocks for sequential processing, with each FE_Block performing additional feature extraction. This sequential processing not only reduces the total number of parameters and computational load but also enriches the feature representation. The outputs from the n FE_Blocks are then concatenated with the other preserved part of the split feature map. This approach integrates multi-level feature information while retaining as much of the original feature data as possible. The concatenated feature map has (n+2)*c channels. Finally, a subsequent convolutional layer reduces the number of channels to c2, ensuring that the output feature map meets the requirements of subsequent processing stages. The FE_C2f module thus achieves efficient use of computational resources and enhances the feature map’s informational richness.

## Experiment

### Dataset

**Fig 9:**
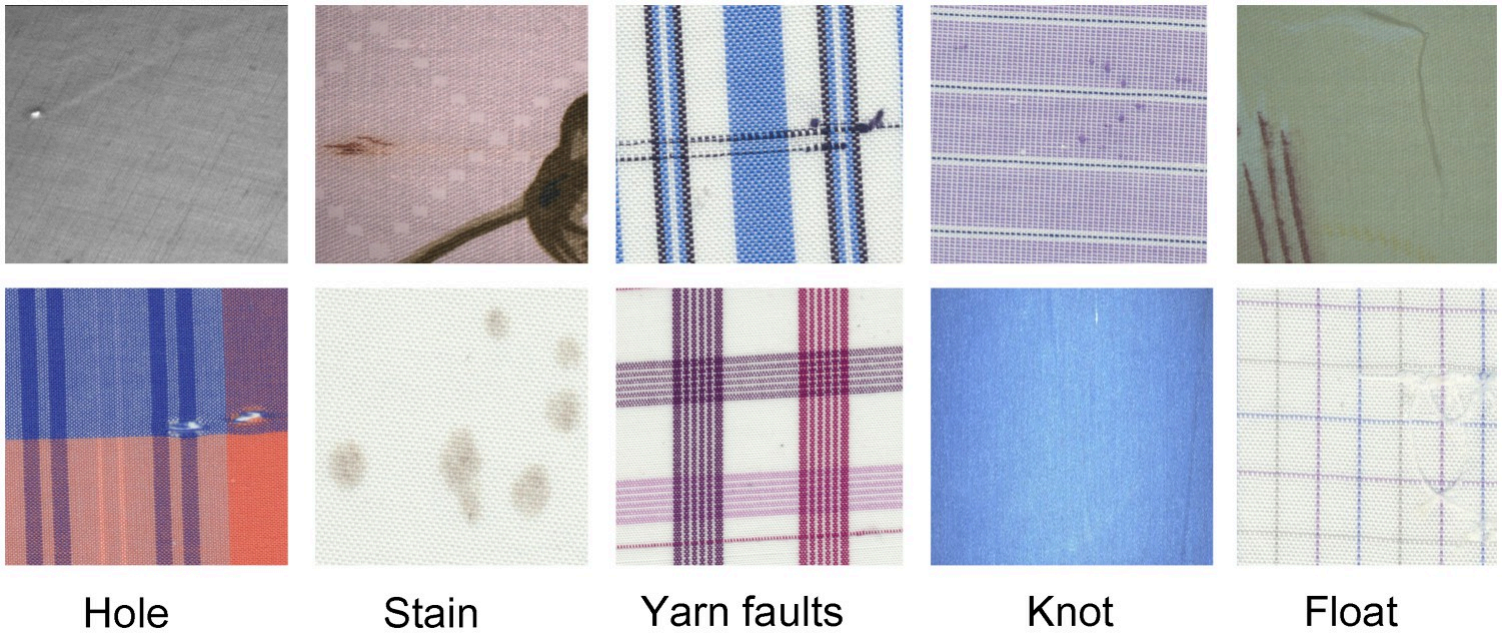
Fabric defect samples.

The experimental dataset was sourced from multiple places, including fabric images from the TILDA database, images provided by the Ali platform, and a textile company in Guangdong. Originally, the dataset comprised 3,575 images. Through preprocessing techniques like scaling, rotation, flipping, and random color adjustments, the sample set was expanded to 5,022 images. The defective fabric images in the dataset are classified into five categories: Hole, Stain, Yarn faults, Knot, and Float. Hole include cuts and punctures, Stain include oil and color stains, and Yarn faults include broken yarn and yarn with slubs. Examples of some fabric defects are shown in Fig 9.

### Environment

The experimental environment configuration is shown in Table 1. Unified parameters were used during the training phase: an initial learning rate of 0.01, momentum of 0.937, weight decay of 0.0005, batch size of 16, 300 training epochs, and an input image size of 640 × 640.

**Table 1:** Experimental environment configuratio.

Category	Version Number
CPU	12vCPUIntel(R)Xeon(R)Platinum8255CCPU@2.50GHz
GPU	RTX3080(10GB)
Deployment Environment	Python3.8(ubuntu20.04)
Deep Learning Framework	PyTorch1.11.0
Acceleration Framework	CUDA11.3

### Evaluation metrics

To evaluate the effectiveness of the proposed algorithm for fabric defect detection, this paper uses precision (P), recall (R), and mean average precision (mAP) as performance metrics. Model complexity is measured by parameter count, and detection speed is measured by computational cost (FLOPs). The mAP@0.5 is used as the primary performance metric, representing the average precision (AP) when the Intersection over Union (IoU) threshold is set to 0.5. The evaluation metrics are calculated using the following formulas:

$$\begin{array}{c} P=\frac{TP}{TP+FP} \end{array}$$ (5)

$$\begin{array}{c} R=\frac{TP}{TP+FN} \end{array}$$ (6)

$$\begin{array}{c} AP=\int_{0}^{1}P\left(R\right)d_{R} \end{array}$$ (7)

$$\begin{array}{c} mAP=\frac{\sum_{i=1}^{K}AP_{i}}{K} \end{array}$$ (8)

Where P is precision; R represents recall; TP is the number of true positive samples correctly identified; FP is the number of false positive samples; FN is the number of false negative samples. The mAP is calculated by averaging the AP of each category, where K represents the number of categories.

## Comparison experiments with different attention mechanisms

The mainstream attention mechanisms include the Channel Attention Mechanism (SE) [41], the Convolutional Block Attention Mechanism (CBAM) [42], the Efficient Channel Attention Mechanism (ECA) [43], and the Dual-Path Routing Attention Mechanism BRA [44]. To validate the effectiveness of the CPCA attention mechanism, it is compared with SE, CBAM, ECA, and BRA attention mechanisms. The experiments are conducted under the same parameters. The experimental results are shown in Table 2 below, with the best results bolded.

**Table 2:** Comparison of different attention mechanisms.

Model	P	R	mAp@0.5
YOLOv8n	83.8	81.5	86.5
YOLOv8n+SE	85.0	79.7	87.4
YOLOv8n+CBAM	85.5	83.2	87.9
YOLOv8n+ECA	85.9	82.2	87.6
YOLOv8n+BRA	84.1	81.1	86.3
YOLOv8n+CPCA	**86.1**	**83.4**	**87.9**

**Fig 10:**
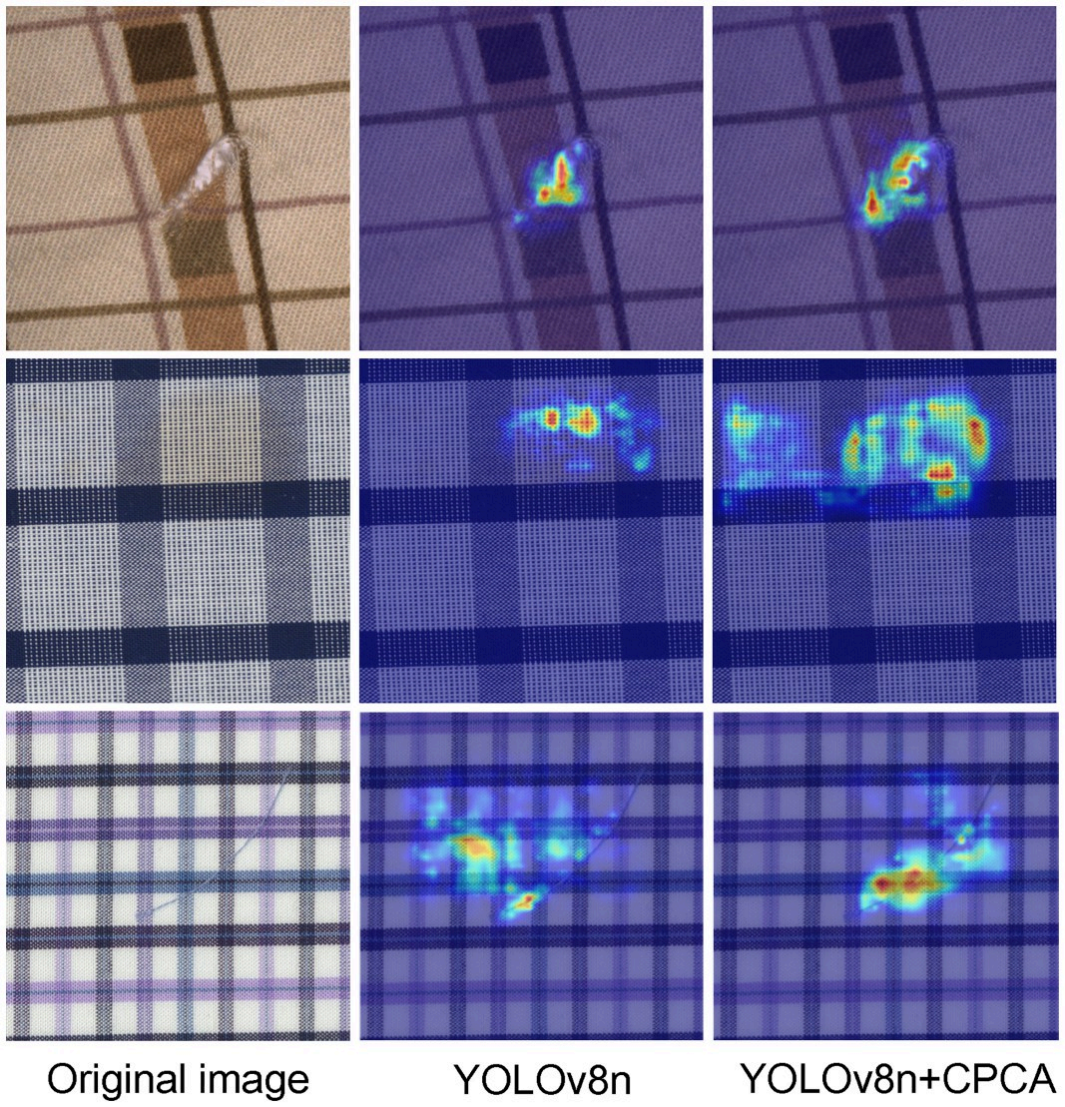
Comparison of CPCA attention mechanism effects.

As illustrated in the Table 2, incorporating the attention mechanism has enhanced the network’s defect localization capability, significantly improving the overall detection accuracy for fabric defects. The data indicate that after adding any type of attention mechanism, the model shows improvement in the mAP@0.5 metric, demonstrating that attention mechanisms indeed strengthen the model’s ability to recognize fabric defects. Among all the listed attention mechanisms, the CPCA attention mechanism exhibits the most significant overall improvement, with a P value reaching 86.1%, a 1.3% increase over the baseline model; a R value reaching 83.4%, a 1.9% increase over the baseline model; and an mAP@0.5 value reaching 87.9%, a 1.4% increase over the baseline model. Except for the SE attention mechanism, all other attention mechanisms show improvements in precision and recall. Based on the analysis, this paper adopts the CPCA attention mechanism inserted after the backbone network’s SPPF layer to enhance defect localization accuracy and improve fabric defect detection performance. To visually demonstrate the improvement of the CPCA attention mechanism in this paper, images are randomly selected from the dataset for feature visualization processing. Heatmaps (where the intensity of colors indicates the model’s focus, with warmer colors indicating higher focus and cooler colors indicating lower focus) before and after integrating the CPCA attention mechanism are generated for comparison. The specific effect is shown in Fig 10.

From Fig 10, it’s clear that the original YOLOv8 network doesn’t accurately localize fabric defects. However, after introducing the CPCA attention mechanism, the areas of focus become more concentrated and prominent. Especially in the yellow and red regions, the model can more precisely localize defects and show stronger responses to defect features. The introduction of this attention mechanism reduces the influence of background information on fabric detection, enabling the model to focus more on the critical parts of defects, thereby enhancing the accuracy of fabric detection in complex scenes.

### Ablation experiment

To validate the impact of each improvement module of the YOLOv8 model proposed in this paper on fabric defect detection performance, ablation experiments were conducted under unified experimental conditions and parameter settings to evaluate the effectiveness of each improvement module. The experimental results are shown in Table 3 below, where ‘✓’ indicates the use of the corresponding module, and the best results are bolded. The baseline network YOLOv8n is denoted as Model A. Model B replaces the C2f module with the DSC_C2f module in the backbone network of Model A. Model C embeds the CPCA module after the SPPF layer at the end of the backbone network of Model A. Model D replaces the C2f module with the FE_C2f module in the neck network of Model A. Model E simultaneously uses the DSC_C2f module and the CPCA attention mechanism on Model A. Model F uses the CPCA attention mechanism and the FE_C2f module on Model A. Model G uses the DSC_C2f module, the CPCA attention mechanism, and the FE_C2f module on Model A, performing ablation experiments on the baseline network YOLOv8n.

**Table 3:** Ablation experiments of different modules.

Model	DSC_C2f	CPCA	FE_C2f	P	R	mAP@0.5	Parameters	FLOPs
A				83.8	81.5	86.5	3.0M	8.1G
B	✓			84.7	83.5	88.2	3.4M	8.3G
C		✓		86.1	83.4	87.9	3.1M	8.3G
D			✓	84.3	82.9	87.0	**2.7M**	**7.7G**
E	✓	✓		86.0	83.2	88.8	3.5M	8.7G
F		✓	✓	87.1	83.4	88.7	2.9M	7.9G
G	✓	✓	✓	**87.3**	**83.7**	**89.4**	3.3M	8.4G

From Table 3, compared to the original Model A, in Model B, the P value increased by 0.9%, the R value increased by 2%, and the mAP@0.5 value increased by 1.7%. This improvement is attributed to the ability of DSConv to adjust the convolution kernel shape to suit changes in input features, allowing the network to capture slender and weak fabric defects across different scales and orientations. Compared to the original Model A, in Model C, the P value increased by 2.3%, the R value increased by 1.9%, and the mAP@0.5 value increased by 1.4%. This is because fabric defects vary in size and have multiple scales, and the CPCA attention mechanism employs multi-scale depth separable convolution modules to construct spatial attention, enhancing detection capabilities. Compared to the original Model A, in Model D, the P value, R value, and mAP@0.5 value increased slightly, while the number of parameters and computational complexity decreased significantly. This indicates that the FE_C2f module effectively reduces the model’s complexity and computational resource consumption while improving performance. Compared to the original Model A, in Model E, the P value increased by 2.2%, the R value increased by 1.7%, and the mAP@0.5 value increased by 2.3%. In Model F, the P value increased by 3.3%, the R value increased by 1.9%, and the mAP@0.5 value increased by 2.2%. In Model G, the P value increased by 3.5%, the R value increased by 2.2%, and the mAP@0.5 value increased by 2.9% compared to the original Model A. These ablation experiments demonstrate the effectiveness of the improvement modules proposed in this paper on the dataset.

### Model comparison experiments

#### Comparison of different models

To verify the performance of the improved model proposed in this paper, a series of classic models were selected for comparative experiments. The experimental results are shown in Table 4, with the best results highlighted in bold.

**Table 4:** Model comparison experiments.

Model	P	R	mAp@0.5	mAP@0.5:0.95
Faster RCNN	73.4	72.1	79.3	40.1
YOLOv5	78.8	80.7	84.9	43.5
YOLOv6	74.2	68.6	75.6	39.1
YOLOv7-tiny	81.9	72.2	79.5	39.6
YOLOv8n	83.8	81.5	86.5	46.9
DCFE-YOLO	**87.3**	**83.7**	**89.4**	**49.0**

Table 4 vividly demonstrates that the improved model surpasses its counterparts across four pivotal metrics,inclouding Precision(P), Recall(R), mAP@0.5, and mAP@0.5:0.95. Compared with the Faster RCNN algorithm, the improved model achieved more than a 10% increase in P, R, and mAP@0.5 values. Compared to the YOLOv5 algorithm, the enhanced model achieves an 8.5% increase in P, a 3% increase in R, a 4.5% increase in mAP@0.5, and a 5.5% increase in mAP@0.5:0.95. Relative to the YOLOv6 algorithm, the P value increases by 13.1%, the R value increases by 15.1%, the mAP@0.5 value increases by 13.8%, and the mAP@0.5:0.95 value increases by 9.9%. When compared to the YOLOv7-tiny algorithm, the P value increases by 6.4%, the R value increases by 11.5%, the mAP@0.5 value increases by 9.9%, and the mAP@0.5:0.95 value increases by 9.4%. Relative to the baseline model YOLOv8n algorithm, the P value increases by 3.5%, the R value increases by 2.2%, the mAP@0.5 value increases by 2.9%, and the mAP@0.5:0.95 value increases by 2.1%.

In conclusion, the improved DCFE-YOLO algorithm outperforms other models across precision, recall, mAP@0.5, and mAP@0.5:0.95. While maintaining high precision, the improved model detects more defects and exhibits stable detection performance across different IoU thresholds, making it better suited for fabric defect detection tasks.

#### Comparing the detection effect of DCFE-YOLO network with theoriginal network model

To evaluate the enhancement in fabric defect detection performance, comparative experiments were conducted between the improved model and the baseline model YOLOv8n. The average detection precision across different defect categories before and after enhancement is compared in Table 5.

**Table 5:** Comparison of mAP@0.5 for different defect categories.

Defect type	YOLOv8n	DCFE-YOLO
All	0.865	**0.894**
Hole	0.876	**0.912**
Stain	0.878	**0.895**
Yarn faults	0.894	**0.938**
Knot	0.773	**0.795**
Float	0.903	**0.933**

The comparison results presented in Table 5 illustrate that the enhanced model demonstrates superior detection performance across all defect categories compared to YOLOv8n, with an average category precision increase of 2.9% over the baseline. Notably, there are improvements observed in all five categories within the dataset, with yarn defects experiencing the most significant enhancement, showing a 4.4% increase over the original model. Selvage defects, being relatively smaller in size compared to other categories, pose challenges in detection, yet the improved model showcases a 2.2% improvement over the baseline model. These findings solidify the effectiveness of the enhanced model in improving detection precision, both globally and locally, compared to the baseline model.

**Fig 11:**
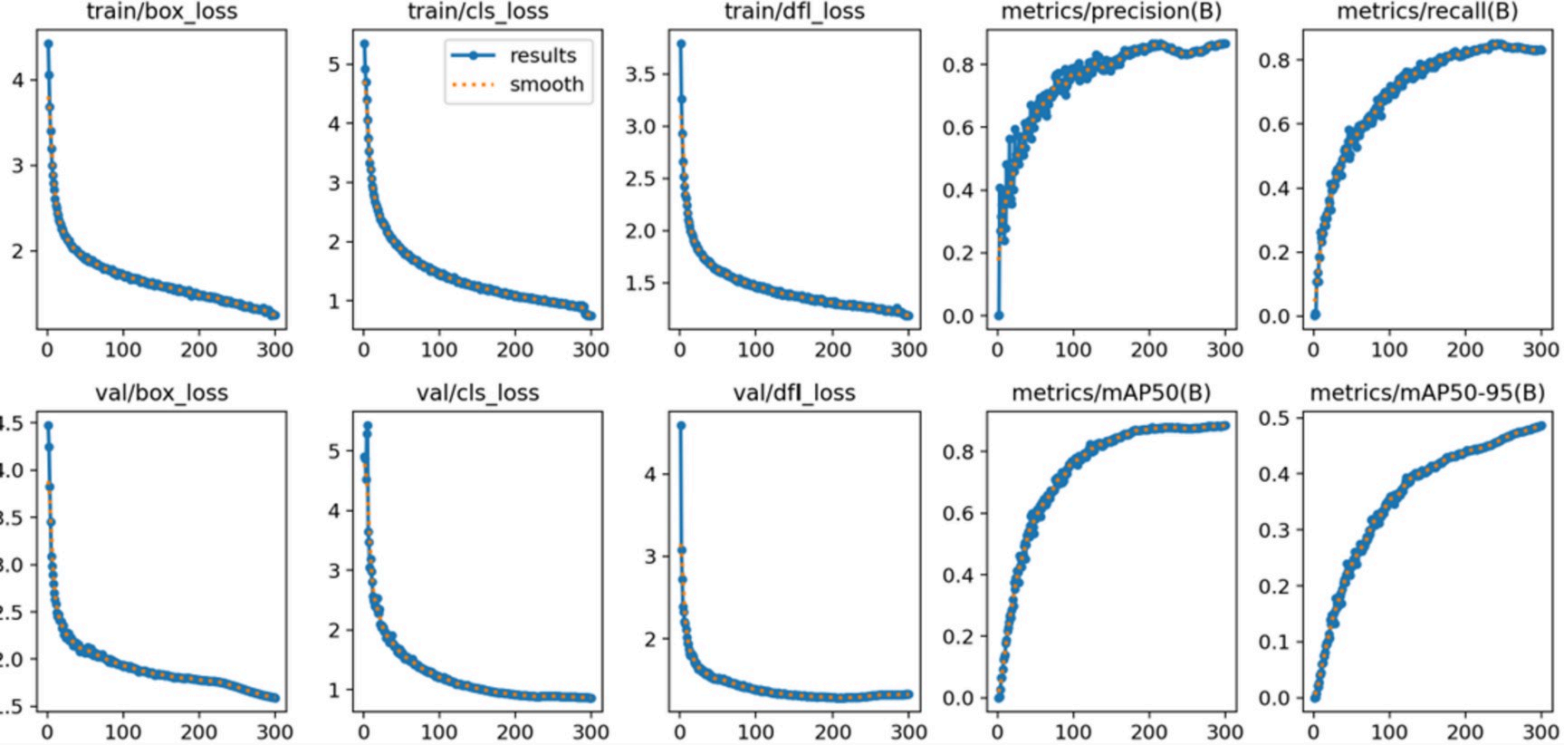
Verification of the fitting degree between the improved model and the data.

This paper applies data augmentation to the existing dataset and implements an early stopping mechanism to mitigate overfitting. Additionally, it increases the number of training epochs and enhances the model’s feature extraction capabilities to address potential underfitting issues. To evaluate the model’s fit to the data, the training loss, validation loss, and accuracy are analyzed across multiple training epochs. In the case of overfitting, the training loss decreases while the validation loss begins to rise after a certain number of epochs. Conversely, if the model is underfitting, both training and validation losses remain elevated, indicating that the model fails to capture the underlying patterns in the data. According to the results presented in Fig 11, the model demonstrates neither overfitting nor underfitting, suggesting that it fits the data effectively and can generalize well to unseen data.

**Fig 12:**
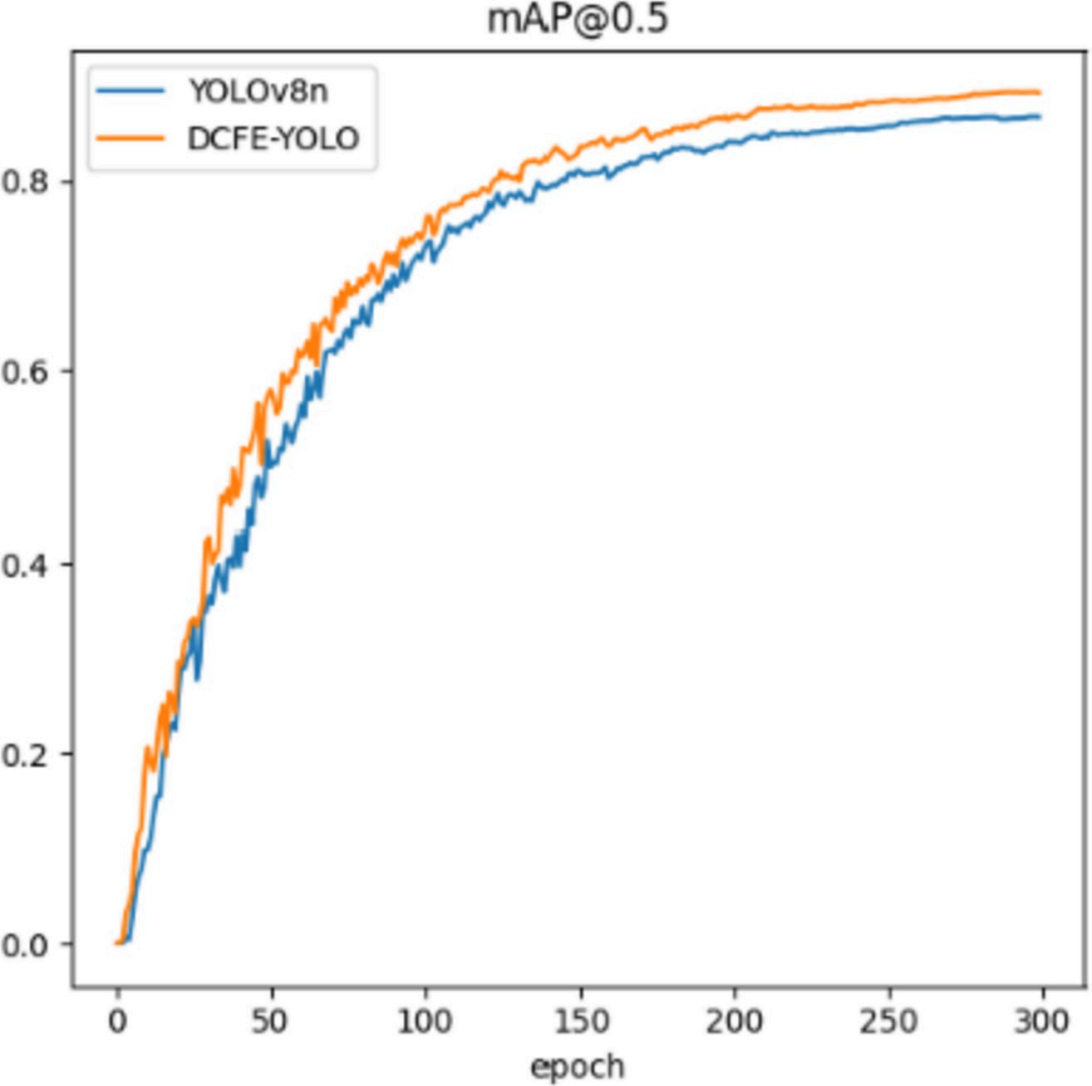
Before and after improvement mAP@0.5 comparison of indicators.

Fig 12 provides a visual comparison of the mAP@0.5 values before and after the model enhancement under the same parameter settings. Initially, during training, both curves exhibit a similar growth trajectory. However, as training progresses, particularly around the 50th iteration, the mAP values of the improved model begin to outperform those of the original model. This trend becomes more pronounced as training nears 300 iterations. Following 300 iterations of iterative training, the improved model demonstrates a more stable convergence trend during training, achieving higher detection accuracy. Its detection performance notably surpasses that of the original model.

**Fig 13:**
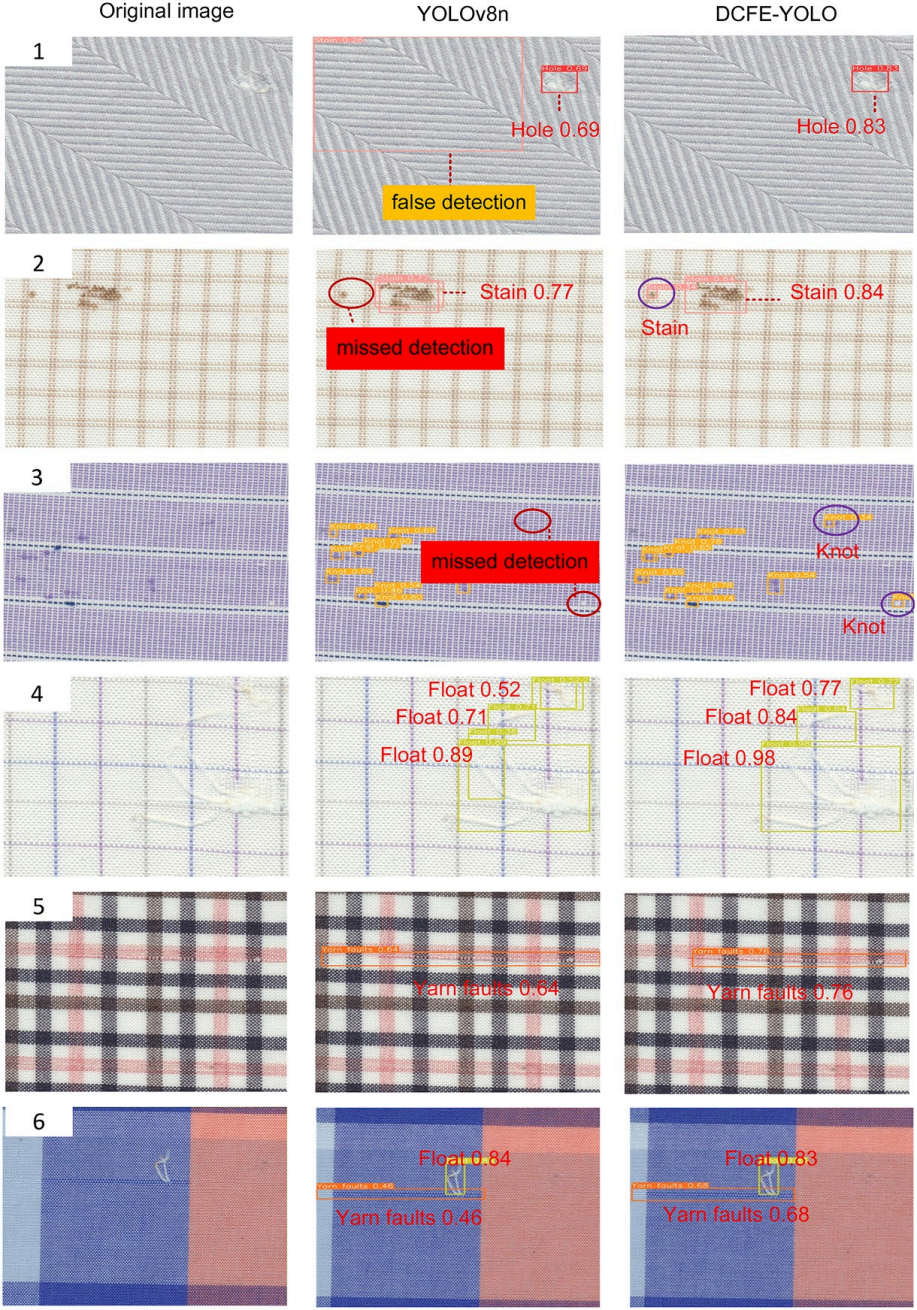
Comparison of results for fabric defect detection before and after improvement.

To offer a more illustrative demonstration of the effectiveness of the proposed enhancement algorithm, fabric defect images of different defect categories under various backgrounds were randomly selected for testing. The fabric defect detection results are depicted in Fig 13. Groups 1-5 in Fig 13 correspond to the detection of five single categories of defects: holes, stains, yarn defects, selvage defects, and floating threads, respectively, while Group 6 represents the detection of multiple defect categories. The experimental outcomes reveal that the improved model excels over the original model in detecting fabric defects against complex backgrounds such as twill, plaid, stripe, and plain fabrics. In the defect detection of images in Groups 1-3, the baseline YOLOv8n model exhibits partial defect omissions and misidentification of background texture information. However, in the detection results of the DCFE-YOLO model, all defects are accurately detected, effectively mitigating omission and misidentification issues. In the defect detection of images in Groups 4-6, compared to the original YOLOv8n model, the improved model demonstrates higher precision and more precise defect localization in fabric defect detection. The performance of the improved model surpasses that of the original model for fabric defect detection tasks.

## Conclusion

In order to further enhance the precision of fabric defect detection, this paper proposes an upgraded YOLOv8n fabric defect detection algorithm. In the C2f layer of the backbone network, a dynamic snake-shaped convolution is introduced to intensify focus on subtle defects, thereby bolstering feature extraction capabilities. Post the SPPF layer in the backbone network, a CPCA attention mechanism is incorporated to pinpoint defect areas through the utilization of multi-scale depth-wise separable convolutions and dynamic weight distributions. Within the feature fusion network, PConv and EMA attention mechanisms are embedded into C2f to enhance detection precision while simultaneously reducing parameter count and computational overhead. Experimental findings demonstrate that the performance of the improved YOLOv8 detection method surpasses that of other conventional detection methods across all evaluation metrics. Compared to the original model, the enhanced model exhibits a 3.5% increase in P, a 2.2% increase in R, and attains a mAP@0.5 value of 89.4%, reflecting a 2.9% improvement over the original network. Considering the multifarious and intricate nature of fabric defect shapes, future endeavors will delve into a more exhaustive exploration of fabric defect categories.
